# Will London Awake?

**Published:** 1904-03-05

**Authors:** Henry Burdett


### WILL LONDON AWAKE?

BY SIR HENRY BURDETT.

In the autumn of 1900 the present Bishop of London, then Bishop of Stepney, preached a sermon at St. Stephen's, Westbourne Park, in which he showed the invaluable work done by clubs in connection with Oxford House and other institutions in his diocese, in reclaiming working lads and girls from the evil influences of the streets, public-houses, and low places of amusement. This sermon led to the formation of the Twentieth Century League, which was incorporated in 1901, and has during the intervening years secured the unity and enlisted the co-operation of the workers amongst boys and girls throughout London. It has succeeded in getting 21 committees formed in the 28 Metropolitan boroughs, and has compiled full statistics of the existing agencies and requirements, on the Oxford House plan, to provide for the whole of the working boys and girls of London aged from 14 years old and under 21. Until this information had been secured and the machinery for developing the work set up, it was decided to delay the appeal for workers and money in sufficient quantity to guarantee, that the whole of the child workers of the Metropolis should be provided with adequate playgrounds, clubs, and the attendant healthy amusements and manual exercises, whereby the present degeneration in the physical stamina of younger Londoners can be arrested and cured without fear of retrogression.

Now that this information has been obtained by the Twentieth Century League the time has come to ascertain whether London through its inhabitants will awake and do its duty. It will be seen that the actual money requirements are relatively small in comparison with the results which can be secured, but I would first impress upon everybody the actual position in regard to this matter as it is to-day. There are in the 28 Metropolitan boroughs 291,725 working boys of the ages mentioned who have no place but the streets to play in with the exception of 25,912 of that number, who are members of one of the 465 boys' clubs, of the right kind and under efficient management, already in existence. Put in another way, out of every 628 boys the existing clubs only receive 55 as members, leaving 573 boys, or 91 per cent. of the total, without the means of healthy recreation and amusement of any kind except the streets. So, too, out of 324,905 working girls only 22,079 are members of the 267 girls' clubs. That is to say, that out of every 1,217 girls, the girls' clubs only receive 83 as members, leaving 1,134 girls, or 93 per cent. of the total without the means of healthy recreation or amusement except the streets.

The foregoing figures give the actual position as it is to-day. Mr. Douglas Eyre (Oxford House), the Chairman of the Executive Committee, has properly laid it down that no funds entrusted to the League shall be expended except upon two conditions: (1) That the club or playing field, or other organisation shall be conducted upon the lines which experience shows will secure the best results; (2) that every club under the auspices of the League shall be directly under the personal charge and management of devoted men and women who have learned how to obtain the best results, and who are prepared to devote themselves and their time to the necessary extent to secure them. If, therefore, sufficient workers and adequate funds are now provided by the heads of families and the more thoughtful of the unmarried men and women amongst us, in the course of the next few years London may be made the happiest place possible for working boys and girls, the stamina of the whole of the rising generation may be raised to the highest point of development, and the moral effects upon the life of the inhabitants must be incalculable.

As to the ways and means, I am anxious to induce at least one lady and one gentleman of means to take charge of the girls and boys respectively in each of the 28 metropolitan boroughs, by agreeing to supply the initial cost of providing the buildings and playing fields which may be necessary to make it possible to accommodate all the working boys and girls who are at present in want of such provision, to enable them to become members of a club or society organised on the Oxford House or a similar system. From all I can ascertain, I think that £1,000 each should prove sufficient at the outset. I would suggest that the buildings and playing fields should be known by the name of the donors of the initial cost, and I cannot but believe that there must be many more than 28 gentlemen and 28 ladies, resident in the Metropolis, who would each be glad to become responsible for so relatively small a sum as £1,000, in view of all the good it might accomplish. This would, of course, be but the commencement of a movement which would, I feel certain, soon be taken up, when the results were published, by a sufficient number of others. The ideal arrangement would be for a few, at any rate, of the pioneer donors to do much more than this, by volunteering to become responsible for everything necessary to complete the equipment of the Metropolitan Borough with which they might decide to identify their name. We want in each borough someone with means who will be prepared to provide the necessary clubs in the right localities, so as to meet exactly the requirements of each, on an organised plan like that which has found expression in the A.B.C. bread shops in various parts of London. If anyone wishes to know the actual capital sum required for one club, or one particular metropolitan borough, I will do my best, if asked, to supply the information.

Of course, when the capital outlay is forthcoming for the Club, it will be necessary to secure such an income for its maintenance as, with the subscriptions of the members, will suffice to maintain it in a high state of efficiency. The Princess of Wales, whose love for children is proverbial, seems to me, as the head of her own household, to have shown the way by her gift of ten guineas. There are in the county of London over one million families or separate occupiers, and my hope is that every well-to-do family, and every well-to-do bachelor, will spontaneously decide to contribute £1 a year towards the maintenance of a boys' or girls' club and playing-field in the metropolitan borough in which they reside. Of course, where the love of children is deepest—and where does it not prevail amongst us?—there the contribution of the family will be largest. I take it that at least one-third of the million householders could easily send £1 every year to the Twentieth Century League, and many of them could, of course, send far more. If one-twentieth will do so, I believe the work can be well and thoroughly done.

If, therefore, the example of her Royal Highness appeals, as I believe it will appeal, to the fathers and mothers of London, then I make bold to think that the response will be so adequate as to testify that London is at length awake to the requirements of its young folk, and that we may all of us sleep at nights with a feeling of thankfulness to the Bishop of London as the father of the Twentieth Century League, and be grateful for the opportunity of co-operating to secure the regeneration of the younger citizens amongst us.

Most important of all, we want a large number of men and women who are fond of children and outdoor amusements of all kinds, to give personal service by offering themselves for training in the management of boys' and girls' clubs. I hope it may be possible to afford them facilities to gain experience at the existing clubs, which will soon render them efficient workers on their own account in connection with the new clubs and organisations, which, we hope, will ultimately be set up in sufficient numbers in every one of the Metropolitan boroughs. We want, also, six men of business to give a little time as members of the Finance Committee.

The present is surely the moment for London to awake, for we have it on the authority of Mr. W. R. M'Connell, K.C., the Chairman of the South London Sessions, that "the reign of terror created"—at the time the Twentieth Century League was instituted—"by roaming gangs of Hooligans seems to have passed away, and for the present a more peaceful disposition appears to have fallen upon the people." Let us, therefore, as citizens worthy of the greatest city in the world, do our duty to the boys and girls of the humbler classes in this matter by providing them with an outlet for their energies which must improve their physical and moral condition, and so perpetuate their present peaceful disposition.

I shall be glad to answer any questions, to receive the names of workers, and to acknowledge any contribution which may be sent to me direct: or contributions may be sent to the Treasurer, Twentieth Century League, the Hon. John Biddulph, Messrs. Cox, Biddulph and Co., 43 Charing Cross, S.W.

